# Hybrid Bio-Based Silicone Coatings with Anti-adhesive Properties

**DOI:** 10.3390/ma16041381

**Published:** 2023-02-07

**Authors:** Maria Nowacka, Anna Kowalewska, Anna Rygala, Dorota Kregiel, Witold Kaczorowski

**Affiliations:** 1 Centre of Molecular and Macromolecular Studies, Polish Academy of Sciences, Sienkiewicza 112, 90-363 Łódź, Poland; 2 Department of Environmental Biotechnology, Faculty of Biotechnology and Food Sciences, Lodz University of Technology, Wólczańska 171/173, 90-924 Łódź, Poland; 3 Institute of Materials Science and Engineering, Lodz University of Technology, Stefanowskiego 1/15, 90-924 Łódź, Poland

**Keywords:** functionalized polysiloxanes and polysilsesquioxanes, eugenol, linalool, surface structure, antimicrobial materials

## Abstract

Hybrid polysiloxanes and polysilsesquioxanes grafted with naturally occurring bioactive phytochemicals: eugenol and linalool, were synthesized and investigated with regard to their structure and properties. The two series of materials, differing in the type of inorganic structure and the content of active groups, were coated onto the surface of glass plates, and their antibiofilm activities against bacteria *Aeromonas hydrophila* were assessed by luminometry and fluorescence microscopy. Bioactivity was correlated with specific properties of the hybrid coatings (chemical structure, surface free energy and adhesiveness). The functionalized polysilsesquioxanes exhibited the most favorable anti-adhesive effects. Cell adhesion after 6 days of incubation, expressed as RLU/cm^2^, was significantly reduced (44 and 67 for, respectively, Z-E-100 and Z-L-100, compared to 517 for the control glass carrier). The surface stickiness of polysiloxane films deteriorated their anti-adhesion properties, despite the presence of a large amount of bioactive species.

## 1. Introduction

Polysiloxanes (silicones) are an important class of inorganic polymers that have found many applications in industry and households and are also used for medical and healthcare purposes. Silicones are characterized by high temperature and oxidative stability, excellent low-temperature flexibility, high resistance to weathering and many chemicals [1,2], as well as biocompatibility, sterilization tolerance, bio-durability and hemocompatibility [3,4]. Non-polar side groups grafted onto siloxane backbones reduce the critical surface tension of silicones and make them capable of wetting most surfaces. Although the intrinsic hydrophobicity of silicones [5] makes them attractive habitats for bacterial settlement and biofilm growth [6,7,8], some specific modifications can lead to improvements in their antimicrobial properties and resistance to biofilm formation [9,10,11,12]. Hybrid materials with activity aimed at killing microorganisms are often based on quaternary ammonium salts, which show quite high toxicity [13]. Despite the environmental concerns, new biocompatible organosilicon compounds bearing quaternary ammonium groups were reported as safe for applications in dentistry, e.g., as disinfectants or medications [14,15] and coating materials capable of killing bacteria on contact [16,17,18]. Antimicrobial superhydrophobic and superoleophobic coatings containing silane quaternary ammonium salts were also used for silk protection [19]. Nevertheless, concerns for the environment have prompted the development of other solutions not based on quaternary ammonium salts but aimed at long-term antimicrobial activity and inhibition of microbial growth. Such strategies have become the focus of research into new generations of environmentally friendly antimicrobial silicones [20,21,22,23].

Modern methods of biofilm control are based on mimicking cell-to-cell communication through the secretion of specific signaling molecules (quorum sensing) [24]. Grafting polysiloxanes with specific chemical molecules that could mimic components of natural quorum-sensing indicators appears to be an attractive alternative. Following this line, several new antibacterial and bacteriostatic silicones have been designed by us and others [25,26,27,28,29,30]. The use of natural substances with antimicrobial properties (for example, 4-allyl-2-methoxyphenol (eugenol) and 3,7-dimethylocta-1,6-dien-3-ol (linalool)) may be another interesting approach to environmentally acceptable antibiofilm materials [31,32,33]. Poor water solubility and volatility of those bioactive phytochemicals limit their use in treating biofilm-related infections. Nevertheless, they were successfully used as active components of bio-based cross-linked networks that exhibited anti-adhesive activity against *Staphylococcus aureus* and *Escherichia coli* [34] and cross-linked polymer nanocomposite “sponges” for the treatment of bacterial biofilms of *E. coli*, *Pseudomonas aeruginosa*, *Enterobacter cloacae* and *S. aureus* [35].

Incorporation of phytochemicals into polymeric materials via covalent bonding permanently links active compounds and prevents their leaching over time. Synthesis of functional silicones by hydrosilylation of eugenol [36,37,38,39,40,41,42] with various Si-H groups containing organosilicon polymers and oligomers was reported. The resulting materials can be used, for example, as anticorrosion additives to commercial epoxy resins [37] and hybrid polyurethanes [42], bio-based silane coupling agents [41] or LED encapsulants [40]. Eugenol and linalool were also used for functionalization of polyhedral silsesquioxanes [38,43]. Antibacterial activity against *E. coli* has been reported for eugenol-grafted MQ resin [39]. However, polymeric siloxanes and polysilsesquioxanes grafted with linalool residues are, to the best of our knowledge, unknown.

**Scheme 1:**
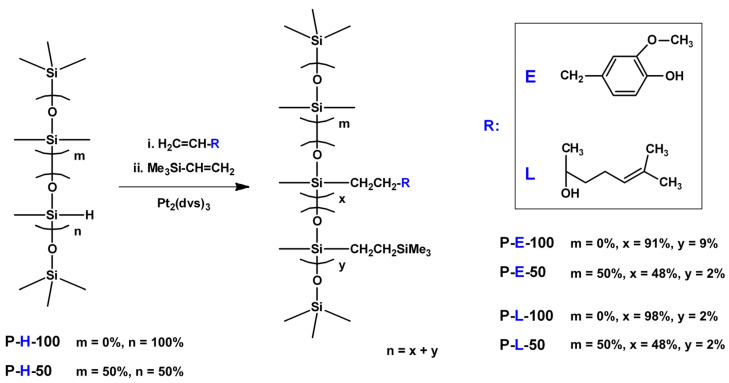
Synthesis of linear siloxane homopolymers and random copolymers with side eugenol and linalool groups (x and y calculated from ^29^Si NMR spectra).

**Scheme 2:**
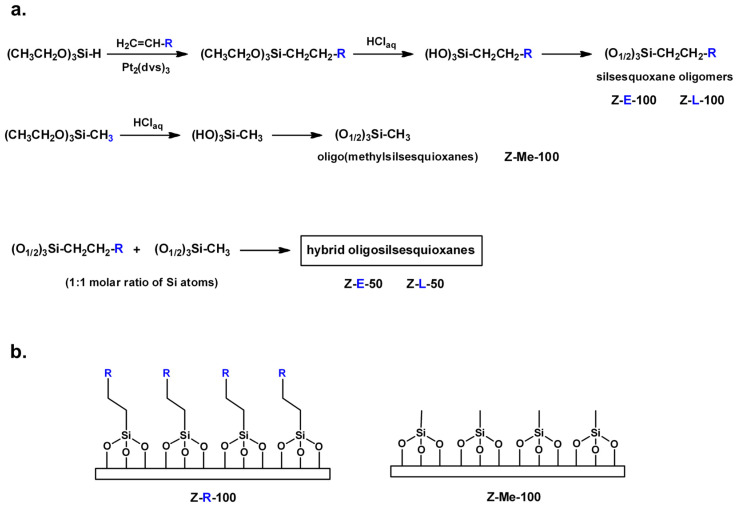
Synthesis of functionalized silsesquioxane sols (**a**) that were used for modification of activated glass plates (**b**) (R as in Scheme 1).

In this report, we present results of comparative studies on polysiloxane (Scheme 1) and polysilsesquioxane-based materials (Scheme 2) grafted with eugenol (E) or linalool (L) residues. They were prepared by hydrosilylation of polyhydrosiloxane and triethoxysilane precursors. The products obtained with HSi(OEt)_3_ were subsequently converted to oligosilsesquioxanes in a sol–gel process.

The materials were tested against water-borne bacterial strain *Aeromonas hydrophila* a KC756842 with strong adhesive properties. *A. hydrophila* is one of the most harmful aquatic pathogens, and environmentally friendly methods of combating this bacterium are highly sought after, including application of phytochemicals [44]. The low solubility in water we have mentioned before hinders their application in the area of aqueous drugs. To solve this problem, eugenol and linalool were used in aqueous environments in the form of Zein nanoparticles [45] or microemulsions [46].

We have found that the hybrid polysilsesquioxane materials grafted both with eugenol and linalool can be used as effective antibacterial hydrophobic coatings against *A. hydrophila*. The exceptional anti-adhesion properties of linalool-modified polysilsesquioxanes should be noted, especially since such materials have not been tested so far against *A. hydrophila* biofilms. Linear polysiloxanes grafted with E and L were not anti-adhesive despite the presence of the bioactive groups. The effect may be related to the surface stickiness of these films. This observation corroborates previous reports indicating that mechanical properties of silicone surfaces can play a very important role in bacterial colonisation due to the conformal overlay of bacterial and soft elastomer surfaces. [47]. Nevertheless, studies of the synthesis and properties of linear E- and L-functionalized polysiloxanes provided useful information that could be translated to other systems, especially cross-linked silicone resins.

## 2. Materials and Methods

The polymeric siloxane precursors: poly(methylhydrosiloxane) (P-H-100; M_nGPC_ = 1800 g/mol, PDI = 2.56) and (50–55% methylhydrosiloxane)-dimethylsiloxane copolymer (random distribution of SiH groups) (P-H-50; M_nGPC_ = 2800 g/mol, PDI = 1.48), both terminated with trimethylsilyl groups, were purchased from ABCR (Karlsruhe, Germany).

Triethoxysilane (95%), eugenol (99%), linalool (97%) and vinyltrimethylsilane (97%) were purchased from Sigma Aldrich (St. Louis, MO, USA) and used as received. Pt^0^/divinyltetramethyldisiloxane complex (Karstedt’s catalyst, 2.1–2.4%Pt, solution in xylene) was obtained from ABCR (Karlsruhe, Germany). Hydrochloric acid (aqueous solution 35–38 wt%) was bought from PPH Stanlab Sp. J., Lublin, Poland.

Solvents used for the synthesis, toluene (Avantor Performance Materials Poland S.A., Gliwice, pure p.a.), dichloromethane (Avantor Performance Materials Poland S.A., Gliwice, pure p.a), ethanol (Avantor Performance Materials Poland S.A., Gliwice, pure p.a) and tetrahydrofuran (THF, Chempur, Karlsruhe, Germany, pure p.a.), were purified according to the literature procedures [48].

### 2.1. Synthesis of Functionalized Polysiloxanes

- **P-E-100:**

P-H-100 (2 g, 0.027 mol SiH) was placed under argon in a round bottom flask containing toluene (30 mL), followed by the addition of eugenol (4.43 g, 0.03 mol). After 15 min at room temperature, Karstedt’s catalyst (30 μL in 3 mL of toluene, 1.41·10^−6^ mol Pt) was added to the reaction mixture.

After about 15 min, the reaction mixture spontaneously warmed up (~40 °C) and became slightly opalescent. The disappearance of SiH groups was followed by FT-IR spectroscopy. After 30 min at RT, 62% conversion of SiH groups was observed. The reaction mixture was then heated to 40 °C, and 92% conversion of SiH groups was noted after 1.5 h.

In the next step, ViSiMe_3_ (0.85 mL, 0.0055 mol) and a new portion of Karstedt’s catalyst (30 μL, 1.41·10^−6^ mol Pt) were added to react with the remaining SiH groups (heating at 40 °C was continued for another 24 h).

The reaction mixture was cooled down to room temperature, and the volatiles were removed under reduced pressure. The crude product was dissolved in CH_2_Cl_2_ and precipitated into petroleum ether twice. The collected precipitate was dried under high vacuum to constant weight. A viscous thick liquid was obtained with 98% yield (6.0 g).

^1^H NMR (CDCl_3_) δ (ppm): 0.12 (-CH_2_SiMe_3_); 0.23 (MeSi(O_2/2_)CH_2_- + -OSiMe_3_); 0.58 (-CH_2_SiMe_3_); 0.72 (MeSi(O_2/2_)CH_2_-); 1.78 (SiCH_2_CH_2_CH_2_-); 2.68 (SiCH_2_CH_2_CH_2_-); 3.87 (OCH_3_); 6.09 (OH); 6.79, 6.99 (Ar)
^13^C NMR (CDCl_3_) δ (ppm): −2.13 (-CH_2_SiMe_3_); 0.02 (MeSi(O_2/2_)CH_2_-); 2.04 (-OSiMe_3_); 17.56 (MeSi(O_2/2_)CH_2_-); 25.52 (SiCH_2_CH_2_CH_2_-); 40.00 (SiCH_2_CH_2_CH_2_-); 55.85 (OCH_3_); 111.36, 114.57, 121.06, 130.75, 134.52, 143.75, 146.61 (Ar)
^29^Si NMR (CDCl_3_) δ (ppm): −22.52 (E-MeSi(O_2/2_)CH_2_-); 3.22 (-CH_2_SiMe_3_); 7.38 (-OSiMe_3_)

- **P-E-50:**

P-H-50 (1 g, 0.0064 mol SiH) was placed under argon in a round bottom flask containing toluene (20 mL), followed by the addition of eugenol (1.05 g, 0.0067 mol). After 15 min at room temperature, Karstedt’s catalyst (16 μL in 1 mL of toluene, 7.51·10^−7^ mol Pt) was added to the reaction mixture.

No spontaneous increase of the reaction mixture temperature was observed. After 30 min at RT, the reaction mixture was heated at 40 °C. The disappearance of SiH groups was followed by FT-IR spectroscopy. After 30 min at RT, 91% conversion of SiH groups was observed, and after another hour of heating at 40 °C, 95% conversion was observed.

ViSiMe_3_ (0.2 mL, 0.0013 mol) and a new portion of Karstedt’s catalyst (16 μL, 7.51·10^−7^ mol Pt) were then added to react with the remaining SiH groups. Heating at 40 °C was continued for another 24 h.

The reaction mixture was then cooled down and concentrated under reduced pressure. The crude product was dissolved in CH_2_Cl_2_ and precipitated into petroleum ether twice. The collected precipitate was dried under high vacuum to constant weight. A viscous, thick liquid was obtained 1.96 g; Y = 98%).

^1^H NMR (CDCl_3_) δ (ppm): 0.125 (-CH_2_SiMe_3_); 0.23 (MeSi(O_2/2_)CH_2_- + -OSiMe_3_); 0.58 (-CH_2_SiMe_3_); 0.72 (MeSi(O_2/2_)CH_2_-); 1.78 (SiCH_2_CH_2_CH_2_-); 2.68 (SiCH_2_CH_2_CH_2_-); 4.02 (OCH_3_); 5.65 (OH); 6.79, 6.99 (Ar)
^13^C NMR (CDCl_3_) δ (ppm): −2.13 (-CH_2_SiMe_3_); −0.09 MeSi(O_2/2_)CH_2_-); 1.36 (Me_2_Si(O_2/2_); 2.02 (-OSiMe_3_); 17.46 (MeSi(O_2/2_)CH_2_-); 25.55 (SiCH_2_CH_2_CH_2_-); 39.36 (SiCH_2_CH_2_CH_2_-); 55.85 (OCH_3_); 111.30, 114.61, 121.12, 134.55, 143.84, 146.63 (Ar)
^29^Si NMR (CDCl_3_) δ (ppm): −22.29 (E-MeSi(O_2/2_)CH_2_-); −21.80 (Me_2_Si(O_2/2_)CH_2_-); 3.20 (-CH_2_SiMe_3_); 7.43 -(OSiMe_3_)

- **P-L-100:**

P-H-100 (2 g, 0.027 mol SiH) was placed under argon in a round bottom flask containing toluene (1 mL), followed by the addition of linalool (4.88 g, 0.030 mol). After 15 min at room temperature, Karstedt’s catalyst (60 μL, 2.82·10^−6^ mol Pt) was added to the reaction mixture.

No spontaneous increase of the temperature of reaction mixture was observed. After 30 min at RT, the reaction mixture was heated at 40 °C. The disappearance of SiH groups was followed by FT-IR spectroscopy, and 91% conversion of SiH groups was observed after 2 h.

ViSiMe_3_ (0.4 mL, 0.0026 mol) and a new portion of Karstedt’s catalyst (15 μL, 7.04·10^−7^ mol Pt) were then added to quench the remaining SiH groups. Heating at 40 °C was continued for another 24 h.

The reaction mixture was then cooled down and concentrated under reduced pressure. The crude product was dissolved in CH_2_Cl_2_ and precipitated into petroleum ether twice. The collected precipitate was dried under high vacuum to constant weight. A viscous, thick liquid was obtained 6.10 g; Y = 94%).

^1^H NMR (CDCl_3_) δ (ppm): −0.01 (-CH_2_SiMe_3_); 0.10 (MeSi(O_2/2_)CH_2_- + -OSiMe_3_); 0.55 (MeSi(O_2/2_)CH_2_-); 1.15 (MeSi(O_2/2_)CH_2_CH_2_C(OH)CH_3_-); 1.49 ((MeSi(O_2/2_)CH_2_CH_2_C(OH)CH_3_-); 1.6–1.8 (-C(OH)(CH_3_) CH_2_CH_2_CH = C(CH_3_)_2_); 2.03 (-C(OH)(CH_3_)CH_2_CH_2_CH = C(CH_3_)_2_); 5.13 (-CH=)
^13^C NMR (CDCl_3_) δ (ppm): −2.27 (-CH_2_SiMe_3_); −0.25 (MeSi(O_2/2_)CH_2_); 2.02 (-OSiMe_3_); 11.23 (MeSi(O_2/2_)CH_2_-); 17.68 (=CCH_3_), 22.66 (-C(OH)(CH_3_)CH_2_CH_2_CH=); 25.65 (-C(OH)(CH_3_)CH_2_CH_2_CH=); 25.82 (=CCH_3_); 35.22 (-SiCH_2_CH_2_C(OH)(CH_3_)-); 41.13 ((-C(OH)(CH_3_)_2_CH_2_CH_2_CH=); 72.92 (SiCH_2_CH_2_C(OH)(CH_3_)-); 124.57 (-CH=); 131.07 (=C(CH_2_)
^29^Si NMR (CDCl_3_) δ (ppm): −21.51 (L-MeSi(O_2/2_)CH_2_-); 3.13 (-CH_2_SiMe_3_); 7.54 -(OSiMe_3_)

- **P-L-50**

P-H-50 (1 g, 0.0064 mol SiH) was placed under argon in a round bottom flask containing toluene (4 mL), followed by the addition of linalool (1.04 g, 0.0067 mol). After 15 min at room temperature, Karstedt’s catalyst (16 μL, 7.51·10^−7^ mol Pt) was added to the reaction mixture.

No spontaneous increase of the temperature of reaction mixture was observed. After 30 min at RT, the reaction mixture was heated at 40 °C. The disappearance of SiH groups was followed by FT-IR spectroscopy, and 92% conversion of SiH groups was observed after 2 h.

ViSiMe_3_ (0.2 mL, 0.0013 mol) and a new portion of Karstedt’s catalyst (16 μL, 7.51·10^−7^ mol Pt) were then added to quench the remaining SiH groups. Heating at 40 °C was continued for another 24 h.

The reaction mixture was then cooled down and concentrated under reduced pressure. The crude product was dissolved in CH_2_Cl_2_ and precipitated into petroleum ether twice. The collected precipitate was dried under high vacuum to constant weight. A viscous, thick liquid was obtained (2.75 g; Y = 92%).

^1^H NMR (CDCl_3_) δ (ppm): −0.01 (-CH_2_SiMe_3_); 0.10 (MeSi(O_2/2_)CH_2_- + -OSiMe_3_); 0.55 (MeSi(O_2/2_)CH_2_-); 1.16 (MeSi(O_2/2_)CH_2_CH_2_C(OH)CH_3_-); 1.47 ((MeSi(O_2/2_)CH_2_CH_2_C(OH)CH_3_-); 1.62 (-C(OH)(CH_3_)_2_CH_2_CH_2_CH=C(CH_3_)_2;_ 1.69 (-C(OH)(CH_3_)_2_CH_2_CH_2_CH=C(CH_3_)_2_); 2.03 (-C(OH)(CH_3_)_2_CH_2_CH_2_CH=C(CH_3_)_2_); 5.13 (-CH=)
^13^C NMR (CDCl_3_) δ (ppm): −2.27 (-CH_2_SiMe_3_); −0.54 (MeSi(O_2/2_)CH_2_); 1.13 (Me_2_Si(O_2/2_)-); 1.77 -OSiMe_3_); 11.13 (MeSi(O_2/2_)CH_2_-); 17.68 (=CCH_3_); 22.66 (-C(OH)(CH_3_)_2_CH_2_CH_2_CH=); 25.57 (-C(OH)(CH_3_)CH_2_CH_2_CH=); 26.02 (=CCH_3_); 34.82 (-SiCH_2_CH_2_C(OH)(CH_3_)-); 40.93 (-C(OH)(CH_3_)_2_CH_2_CH_2_CH=); 72.95 (SiCH_2_CH_2_C(OH)(CH_3_)-); 124.57 (-CH=); 131.07 (=C(CH_2_)
^29^Si NMR (CDC_l3_) δ (ppm): −22.03 (L-MeSi(O_2/2_)CH_2_-; Me_2_Si(O_2/2_)CH_2_-); 2.92 (-CH_2_SiMe_3_); 7.18 -(OSiMe_3_)

### 2.2. Synthesis of Functionalized Triethoxysilanes

- **E-Si(OEt)_3_**

2-methoxy-4-(3-(triethoxysilyl)propyl)phenol was prepared according to the literature procedure [41]. A Schlenk flask was charged under argon with toluene (40 mL) and triethoxysilane (2.4 g, 0.0146 mol). Eugenol (2 g, 0.012 mol) was admixed, and the reaction mixture was then stirred for 5 min at RT. Karstedt’s catalyst (20 μL, 9.39·10^−7^ mol Pt) was subsequently added, and the mixture was heated at 60 °C for 24 h, immediately after mixing all the reagents. The mixture was then cooled to RT, concentrated under reduced pressure, and the resulting crude product was used in the next step (Section 2.3 Preparation of hybrid silsesquioxane sols and coatings).

^1^H NMR (CDCl_3_) δ (ppm): 0.69 (-SiCH_2_-); 1.24 (-OCH_2_CH_3_); 1.73 (-SiCH_2_CH_2_CH_2_-Ar); 2.59 (-CH_2_-Ar); 3.84 (-OCH_2_CH_3_); 5.64 (-OCH_3_); 6.69, 6.84 (Ar)
^13^C NMR (CDCl_3_) δ (ppm): 9.89 (-SiCH_2_-); 17.88 (-OCH_2_CH_3_); 25.03 (-SiCH_2_CH_2_CH_2_-Ar); 38.63 (CH_2_-Ar); 55.27 (OCH_3_); 58.12 (-OCH_2_CH_3_); 111.11, 114.45, 120.83, 133.85, 143.94, 146.63 (Ar)
^29^Si NMR (CDCl3) δ (ppm): −44.89

HRMS: calc. for C_16_H_28_O_5_Si+Na [M+Na] = 351.469, found 351.163

- **L-Si(OEt)_3_**

A Schlenk flask was charged under argon with toluene (42 mL) and triethoxysilane (2.55 g, 0.0156 mol). Linalool (2 g, 0.013 mol) was added, and the reaction mixture was then stirred for 5 min at RT. Karstedt’s catalyst (30 μL, 1.41·10^−6^ mol Pt) was then added, and the mixture was heated at 60 °C for 24 h. The mixture was then cooled to RT, concentrated under reduced pressure, and the resulting crude product was used in the next step (Section 2.3 Preparation of hybrid silsesquioxane sols and coatings).

^1^H NMR (CDCl_3_) δ (ppm): 0.75 (-SiCH_2_); 1.47 (-SiCH_2_CH_2_C(OH)(CH_3_)-) + (-OCH_2_CH_3_); 1.52 (-SiCH_2_CH_2_-); 1.6–1.8 (-C(OH)(CH_3_)CH_2_CH_2_CH=C(CH_3_)_2_); 2.07 (-C(OH)(CH_3_)CH_2_CH_2_CH=); 3.86 (-OCH_2_CH_3_); 5.13 (-CH=)
^13^C NMR (CDCl_3_) δ (ppm): 4.16 (-SiCH_2_-); 17.99 (-OCH_2_CH_3_ + =CCH_3_); 22.72 (C(OH)(CH_3_)CH_2_CH_2_CH=C(CH_3_)_2_); 25.42 (=CCH_3_); 26.94 ((-SiCH_2_CH_2_C(OH)(CH_3_)-); 35.59 (-SiCH_2_CH_2_-); 42.54 (-C(OH)(CH_3_)CH_2_CH_2_CH=C(CH_3_)_2_); 58.87 (-OCH_2_CH_3_); 72.32 (-SiCH_2_CH_2_C(OH)(CH_3_)-); 124.34 (-CH=); 130.89 (=C(CH_2_)_2_)
^29^Si NMR (CDCl_3_) δ (ppm): −28.03 (42%, L-Si(OEt)_3_); −35.11 (43%, L-Si(OEt)_2_(OSi-)); −43.49 (15%, L-Si(OEt)(OSi-)_2_)

### 2.3. Preparation of Hybrid Silsesquioxane Sols and Coatings

**Z-E-100:** Hydrochloric acid (4 μL) was added at room temperature to a stirred mixture of E-Si(OEt)_3_ (0.012 mol) and 0.6 mL of deionised H_2_O. The mixture was stirred for another 5 h. The resulting sol thickened considerably, becoming more viscous and opalescent. The top layer containing water was removed, and the bottom layer was dissolved in THF (3 mL).

**Z-L-100:** Hydrochloric acid (4 μL) was added at room temperature to a stirred mixture of L-Si(OEt)_3_ (0.013 mol) and 0.6 mL of deionised H_2_O. The reaction mixture almost immediately heated up and became homogeneous. At the same time, viscosity of the reaction mixture increased rapidly, and the mixture became opalescent. THF (3 mL) was added to stop this process.

**Z-Me-100:** Distilled water (0.55 mL, 0.030 mol) and concentrated hydrochloric acid (4 μL) were placed in a round bottom flask containing triethoxymethylsilane (2 g, 0.012 mol). After about an hour, the reaction mixture was completely homogeneous.

The surface of glass slides was activated to enhance grafting of sols (Z-type materials). Glass plates were kept overnight in the solution of KOH (25 g) in isopropanol (250 mL) and then washed with copious amounts of distilled water. Thin films on glass slides were prepared by immersing the activated glass slides in solutions containing 30 mL of anhydrous ethanol and 0.5 mL of solutions of Z-X-100 in THF (X = E, L or Me) (prepared as described above). The coated glass slides were then immersed in pure EtOH to remove the excess of sol and left (upright) for drying under ambient conditions for 24 h.

Thin films of binary hybrid sols (Z-E-50 and Z-L-50) were prepared analogously by immersing activated glass plates in solutions composed of a mixture of 0.15 mL of Z-Me-100 and 0.25 mL of Z-E-100 (or 0.25 mL of Z-L-100, respectively) and diluted with anhydrous ethanol (30 mL).

The polysiloxane products (P-type materials) were also used for the preparation of coatings on glass. Thin polymer films of the functionalized polysiloxanes (P-E-100, P-E-50, P-L-100 and P-L-50) were cast from 80 wt% polymer solutions in CH_2_Cl_2_ onto clean glass slides (clean ready to use, Lab Glass) using a slit coating applicator (film thickness 150 μm).

### 2.4. Analytic Methods

#### 2.4.1. Nuclear Magnetic Resonance Spectroscopy (NMR)

Liquid state ^1^H-, ^13^C- and ^29^Si-NMR spectra of the functionalized polysiloxanes were recorded in CDCl_3_ as a solvent on an Avance Neo 400 NMR spectrometer with tetramethylsilane (TMS, Billerica, MA, USA) as the reference.

#### 2.4.2. Fourier Transform Infrared Spectroscopy (FT-IR)

Absorption spectra were recorded for thin films of samples cast on crystal KBr windows using a Nicolet 380 FT-IR spectrometer (Thermo Scientific, Waltham, MA, USA). The spectra were obtained by adding 32 scans at a resolution of 1 cm^−1^. The position of characteristic vibration modes was correlated with literature data [49,50,51,52].

#### 2.4.3. Thermogravimetric Analysis (TGA)

The thermal stability of the studied polymeric materials was studied with a Hi-Res TGA 2950 Thermogravimetric Analyzer (TA Instruments, New Castle, DE, USA) in nitrogen atmosphere (heating rate 10 °C/min, resolution 3, sensitivity 3).

#### 2.4.4. Differential Scanning Calorimetry (DSC)

Phase transitions in the studied samples (native and functionalized polysiloxanes) were detected using a DSC 2500 (TA Instruments, New Castle, DE, USA). Thermograms were taken in N_2_ atmosphere at a rate of 10 °C/min for samples (sealed in aluminum pans) that were cooled from room temperature to −90 °C (5 min isotherm at −90 °C), then heated from −90 ° to 25 °C, cooled again to −90 °C (5 min isotherm at −90 °C) and finally heated to 150 °C. The cooling/heating cycle was repeated, and the temperatures of characteristic phase transitions were taken from the second measurement.

As the lowest temperature that can be reached with the DSC apparatus available is −90 °C, it was not possible to record glass transition for the polymeric precursors (P-H-50 and P-H-100).

#### 2.4.5. Surface Energy Measurements

Surface free energy was estimated at room temperature by contact-angle measurements (sessile drop technique, static contact angles at the film–air interface), as described earlier [53], using deionized water and glycerol (Chempur, pure p.a., anhydrous) as the reference liquids. Contact angles were measured just after the liquid was deposited on the film surface (fixed contact time 1 s). The contact angle values used to assess the free surface energy were the average of five measurements taken on different areas of the same sample. A Rame-Hart NRL contact-angle goniometer equipped with a video camera JVC KYF 70B and drop-shape analysis software was used for the measurements. Surface energies (including their polar and dispersive components) were estimated by the Owens–Wendt method [54].

Uncoated, clean glass was used as a control sample. It was not possible to prepare thin layers of P-H-100 and P-H-50 on glass substrates due to their low density (0.9–1.1 g/mL).

#### 2.4.6. Raman Mapping

Raman signals (spectra and maps) were collected with an InVia Raman microscope (Renishaw plc, Gloucestershire UK). The polysilsesquioxane surfaces were analyzed using a 532-nm laser with 20× objective lens (Carl-Zeiss Jena, Germany), providing approximately 30 mW of laser power at the sample. Single spectra were collected with an exposure time of 7 s in the spectral collection range 600–1800 cm^−1^; 2D Raman maps were collected from 50 × 50 μm areas with 2 µm spatial resolution. The interpolation of spectral maps (for better illustration) and all operations on the data (baseline correction, normalization and intensity at point) were performed using the Wire 5.1 application.

#### 2.4.7. Molecular Modelling

Structures of polysiloxanes functionalized with eugenol and linalool derivatives were constructed on the HyperChem platform [55]. The atomic point charges were calculated using the semi-empirical AM1 method, and geometry was optimized by the molecular mechanics method (using an MM+ force field parameter set with Polak–Ribiere energy minimization algorithms).

### 2.5. Biological Studies

#### 2.5.1. Bacterial Strain

The reference strain of a water-borne bacterial isolate *A. hydrophila* KC756842 with strong adhesive properties, deposited at LOCK Culture Collection, was subcultured on Triptic Soy Agar slants (Merck Millipore, Darmstadt, Germany). The tested microorganism was cultivated at 25 °C for 48 h and then maintained at 4 °C [56].

#### 2.5.2. Assessment of Bacterial Adhesion

Cell adhesion on abiotic surfaces (samples prepared as described in Section 2.3) was evaluated under laboratory conditions. Experiments were conducted using white glass as the control carrier (Star Frost 76 mm × 26 mm, Waldemar Knittel Glasbearbeitungs GmbH, Bielefeld, Germany). Glass carriers and modified carriers were sterilized using UV light (265 nm, 2 h per each side). Sterile carriers (10 mm × 10 mm) were placed in sterile 25 mL Erlenmeyer flasks with 20 mL of 50-fold diluted buffered peptone water (Merck KGaA, Darmstadt, Germany).

The amount of cell inoculum (0.1 mL) was standardized densitometrically (1°McF) using a DEN-1B densitometer (Merck KGaA, Darmstadt, Germany). The bacterial cultures and carriers were incubated at 25 °C with agitation on a laboratory shaker (Unimax 1010 Platform Shaker) at 135 rpm for 6 days. Cell adhesion to the carriers was evaluated using both fluorescence microscopy and luminometry using ATP-free sampling pens and a HY-LiTE2 luminometer (Merck KGaA, Darmstadt, Germany). The luminometric measurements were expressed in RLU/cm^2^ [56]. Adhered bacterial cells were also observed after DAPI staining and using a fluorescence microscope NICON BX41 fitted with a 50× lens and with top illumination of the tested surfaces by an external lamp. Images were captured with a digital camera.

#### 2.5.3. Determination of Antimicrobial Activity of Compounds Used to Create Functional Polymers

The routine antimicrobial susceptibility testing was based on the agar well diffusion method [57]. The agar plate surface was inoculated by spreading 200 µL of the bacterial suspension over the entire TSA on the Petri plates. Then, a hole with a diameter of 5 mm was punched aseptically with a sterile cork borer, and a volume of 10 µL of the tested substance at the desired concentration was introduced into the well. After that, the agar plates were incubated at 25 °C for 24 h. The antimicrobial activity of the tested molecules (eugenol, linalool) was detected by the appearance of the inhibition zone around the well. MIC testing for polymers was not performed for solubility reasons. The inhibition coefficient Q was calculated according to the formula:

Q = inhibition zone diameter [mm]/well diameter [mm]

#### 2.5.4. Statistical Methods

The results of cell adhesion were calculated as the means and standard deviations from three independent tests. Analysis of variance (ANOVA) was used to examine the differences between group means, representing the adhesion results (OriginLab Corporation, Northampton, MA, USA). The results were compared to those for the control samples (glass carriers). Values with letters show statistically significant differences: a, *p* ≥ 0.05; b, 0.005 < *p* < 0.05; c, *p* < 0.005.

## 3. Results and Discussion

### 3.1. Synthesis and Properties of Organosilicon Derivatives of Eugenol and Linalool

#### 3.1.1. Functionalized Polysiloxanes and Oligomeric Silsesquioxanes

**Figure 1:**
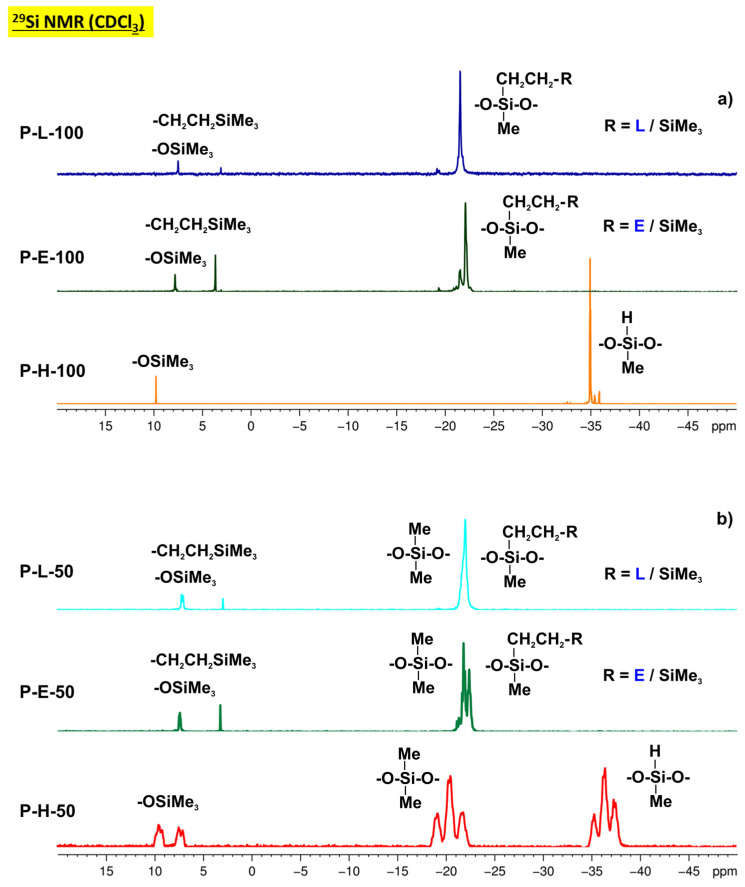
^29^Si NMR spectra of linear polysiloxanes grafted with eugenol and linalool (compared to their precursors P-H-100 (**a**) and P-H-50 (**b**)).

**Figure 2:**
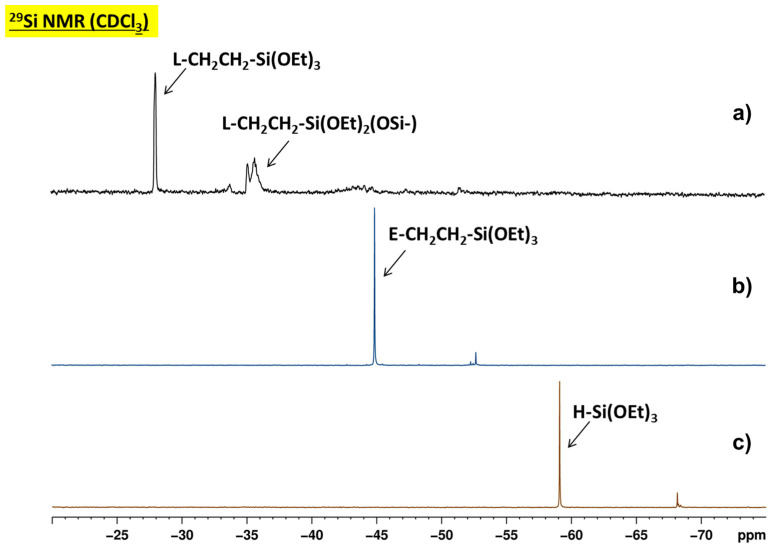
^29^Si NMR spectra of the products obtained by hydrosilylation of linalool (**a**) and eugenol (**b**) with HSi(OEt)_3_ (**c**).

We synthesized two series of organosiloxane materials using Pt^0^/divinyltetramethyldisiloxane complex (Pt_2_(dvs)_3_, Karstedt’s catalyst) as a readily commercially available catalyst for hydrosilylation of eugenol and linalool with polysiloxanes (P-type materials) and HSi(OEt)_3_ (Z-type materials). The structure of the functionalized derivatives was confirmed with ^1^H, ^13^C and ^29^Si NMR spectroscopy (Figure 1, Figure 2 and Figures S1–S4) as well as FT-IR spectroscopy (Figures S5, S6 and Table S1).

Under the conditions used, hydrosilylation of the terminal alkene groups of both organic compounds was found to be the predominant process that obeyed the Farmer’s rule (anti-Markownikov rule). However, in the case of linear polysiloxanes we found that the conversion of Si-H groups was not completed despite a slight excess of the olefins used for the reaction because of the steric hindrance of neighbouring side chains. To stabilize the product, the remaining SiH groups were blocked by the addition to ViSiMe_3_. It was noted that the addition of allyl guaiacol occurred smoothly at room temperature. Heating to 40 °C slightly improved the degree of SiH conversion. In contrast, the addition of linalool to P-H-100 and P-H-50 required heating the reaction mixture (0% conversion of Si-H groups at room temperature).

Although the synthesis of E-Si(OEt)_3_ and L-Si(OEt)_3_ was carried out under similar conditions, only the former one was obtained as a single product. The addition of linalool to HSi(OEt)_3_ resulted in a mixture of L-Si(OEt)_3_ (−28.03 ppm; 42%) and oligomeric species—tentatively identified as L-Si(OEt)_2_(OSi-) (−35.11 ppm; 43%) and L-Si(OEt)(OSi-)_2_ (−43.49 ppm; 15%)—that formed as a result of partial hydrolysis of Si-OEt groups under reaction conditions and subsequent formation of Si-O-Si bonds (Figure 2). Neither isomerization of allyl groups in eugenol, hydrosilylation of sterically hindered 3,7-dimethylolefin in linalool nor O-silylation of the hydroxyl groups in 4-allyl-2-methoxyphenol and 3,7-dimethylocta-1,6-dien-3-ol was noted. These results corroborate the report by Sokolnicki et al. who found that the conversion of eugenol and selectivity of the reaction carried out with Pt_2_(dvs)_3_ depended on the catalyst concentration [41].

#### 3.1.2. Thermal Properties of Polysiloxanes Grafted with Eugenol and Linalool

**Figure 3:**
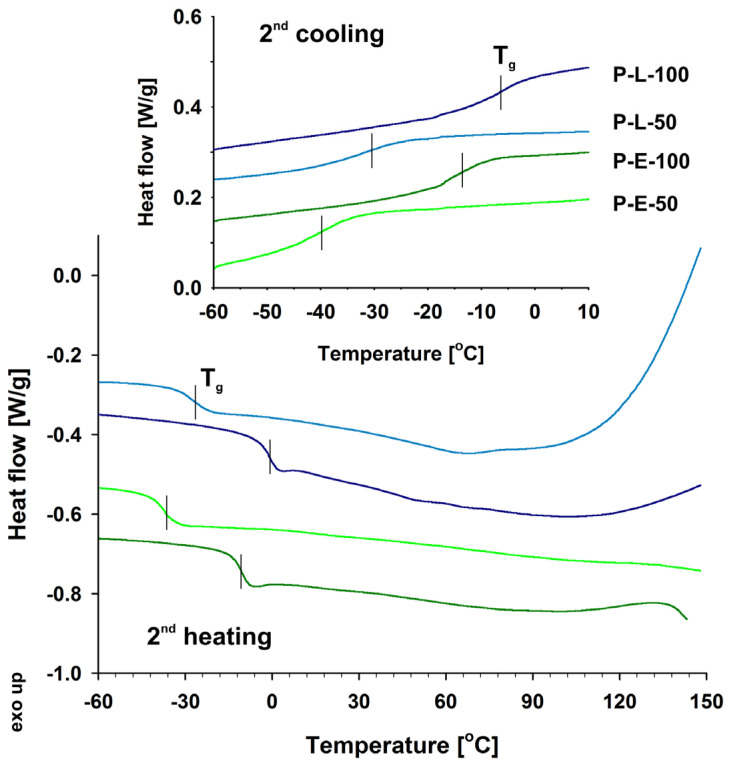
DSC curves (2nd cooling and heating) of the functionalized polysiloxanes.

Calorimetric analysis of the synthesized functionalized polysiloxanes showed (Figure 3) that their characteristic low glass transition temperature (T_g_) is much higher than those reported for their hydrosiloxane precursors (−134 °C and −142 °C for, respectively, P-H-50 and P-H-100 [58]). Moreover, a difference in T_g_ was noted depending both on the type and amount of functional groups grafted to the polysiloxane backbone. Despite the presence of bulky side methoxyphenol groups, polysiloxanes grafted with eugenol exhibited slightly lower temperatures of glass transition that their linalool counterparts. The effect was observed both for homopolymers P-E-100 and P-L-100 as well as copolymers P-E-50 and P-L-50. It can be explained in terms of steric hindrance provided by side 3,7-dimethylocta-6-en-3-ol groups and the presence of hydroxyl and methyl groups situated in the proximity of the polysiloxane chain.

**Figure 4:**
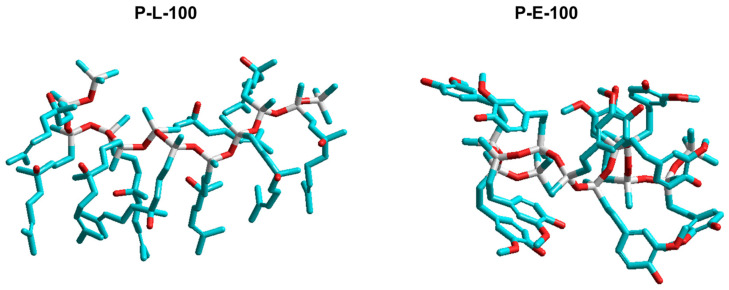
HyperChem calculated structures for P-L-100 and P-E-100 composed of 10 siloxane units.

The structures of functionalized polysiloxanes were constructed on the HyperChem platform (Figure 4). A clear tendency toward cooperative organization of side chains, which can affect the mobility of the polymer and consequently the glass transition process, can be observed for both P-L-100 and P-L-50. In contrast, no propensity toward such organization was found for guaiacol residues. From the models, it appeared that P-E-100 and P-E-50 macromolecules were more likely to form coils due to the high flexibility of the siloxane chain and side group interactions. Nevertheless, a small exotherm that appeared for P-E-100 above 110 °C during the second heating may indicate a predisposition of this polymer to cold crystallization at elevated temperatures.

**Figure 5:**
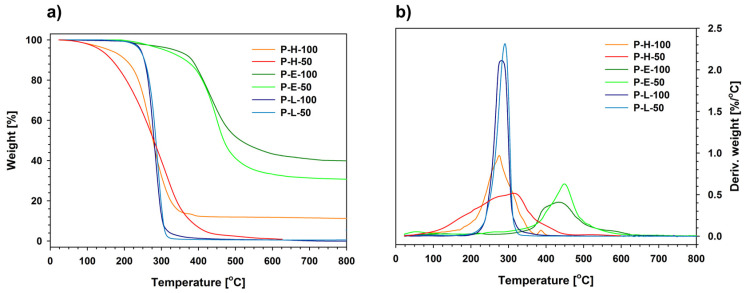
Comparison of TGA curves (N_2_, 10 °C/min) of the studied polysiloxanes (**a**) sample weight decrease and (**b**) derivative of the weight change.

DSC curves of both linalool derivatives showed that the studied samples started to decompose at temperatures higher than 100 °C during the second heating run that was extended to 150 °C. Polymers with side 2-methoxyphenol-4-propyl groups were stable within this temperature range. The differences in thermal stability of the obtained polymeric products were also shown by thermogravimetric analysis (TGA) under a nitrogen atmosphere at a temperature ramp of 10 °C/min (Figure 5). The 5% weight loss temperature (T_d5%_) and peak temperature of weight loss derivative (T_d1_) was determined for all the modified polysiloxanes as well as their inorganic and organic precursors (Table 1). Grafting both eugenol and linalool to siloxane macromolecules significantly increased the temperature range at which such antibiofilm materials could be used, compared to native eugenol and linalool that tend to evaporate rapidly at temperatures close to 100 °C.

Both poly(methylhydrosiloxane) (P-H-100) and (methylhydrosiloxane)-dimethylsiloxane copolymer (P-H-50) start to depolymerize at temperatures close to 100 °C, following the cyclization mechanisms involving chain scission and backbiting [59,60]. Thermal degradation of P-H-100 may be accompanied by partial cross-linking between silanol groups (formed due to the primary chain scission) and the remaining Si–H groups [61]. This process explains slightly larger residue obtained at 500 °C for P-H-100 than for P-H-50.

**Scheme 3:**
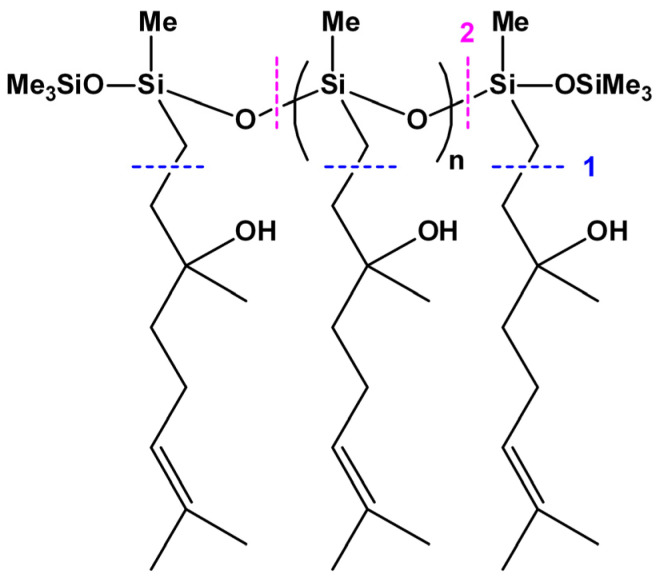
Tentative mechanism of thermal degradation of linalool-functionalized linear polysiloxanes (1: cleavage of C-C bonds in the side chains; 2: depolymerization of the polysiloxane backbone).

Thermal degradation of polysiloxanes grafted with either eugenol or linalool is significantly different from that of their hydrosiloxane precursors. Both P-E-100 and P-E-50 exhibited much higher thermal stability in the nitrogen atmosphere (T_d1_ = 430–450 °C) than their analogues P-L-100 and P-L-50 (T_d1_ = 270–280 °C). The higher number of organic groups in the side chains of P-E-100 and P-L-100 does not appear to increase the thermal resistance compared to copolymers P-E-50 and P-L-50. This effect can be tentatively explained by the cleavage of C-C bonds in the side chains, followed by depolymerization of the resulting poly(methylalkylsiloxanes) (Scheme 3). Similar phenomena occurred during thermolysis of hybrid silsesquioxane–carbosilane materials in an inert atmosphere [49].

This hypothesis is further supported by the equally high thermal stability of both eugenol derivatives that have T_d1_ higher by about 160–170 °C than P-L-100 and P-L-50. The process can be tentatively explained in terms of slow abstraction of ethylguaiacol groups. In addition to the similarity of T_d1_ characteristic of P-E-100 and P-E-50, the ceramic residue left after decomposition of the latter are only slightly lower than those of the homopolymer. A slow decrease of sample weight at 500–700 °C indicates that the subsequent degradation process is not based on the depolymerization of polysiloxanes but involves a range of complex phenomena. We noted a similar increase in the thermal stability of linear polysiloxanes grafted with derivatives of sterically demanding tris(trimethylsilyl)methane, both as terminal [62] and side groups [58]. Thus, the increase in thermal stability of linear polysiloxanes grafted with eugenol and linalool may be related to a reduction in the extent of thermal depolymerization by chain scission.

### 3.2. Surface Structure and Properties of Thin Films Obtained with Functionalized Polysiloxanes and Hybrid Silsesquioxane Sols

#### 3.2.1. Coatings Made of Functionalized Silsesquioxane Sols

Thin polymer films of all P-type materials were coated as 80 wt% solutions in CH_2_Cl_2_ (both homopolymers and copolymers polymers are quasi-hard waxes) onto clean glass slides using a slit coating applicator. The layer thickness of 150 μm was chosen experimentally as optimal, resulting in continuous polymer films. Although the films showed no tendency to de-wet during storage, it should be mentioned that they were tacky. Glass plates with functionalized Z-type oligosilsesquioxanes grafted on the surface were prepared using sols, as described in Section 2.2. The sols were prepared by acid hydrolysis of the functionalized trialkoxysilanes carried out at room temperature. Hybrid sols containing 50% molar amount of methylsilsesquioxane units (Z-E-50, Z-L-50) were formulated by mixing appropriate amounts of Z-E-100 or Z-L-100 with a methyltriethoxysilane-derived sol obtained under similar conditions. The oligosilsesquioxane sols contained SiOH groups, which were used for the formation of covalent bonds with activated glass plates. Thin films of Z-type materials were coated on glass supports by immersion in THF/ethanol solutions of the corresponding oligosilsesquioxanes. After drying under ambient conditions, the coated glass plates were non-sticky and ready for characterization of their anti-adhesion properties and microbiological studies.

The random distribution of functional groups along the polysiloxane chains in the P-R-50 makes any phase separation and formation of clusters enriched in E or L groups completely impossible. It may have been different with the structure of silsesquioxane sols Z-R-50. At the homogenization stage of an acidic sol–gel process, oligomeric silsesquioxane species are already formed due to partial cross-linking of silanols and formation of Si-O-Si bonds [63,64,65]. This fact might affect the structure of Z-R-50 hybrid materials.

**Figure 6:**
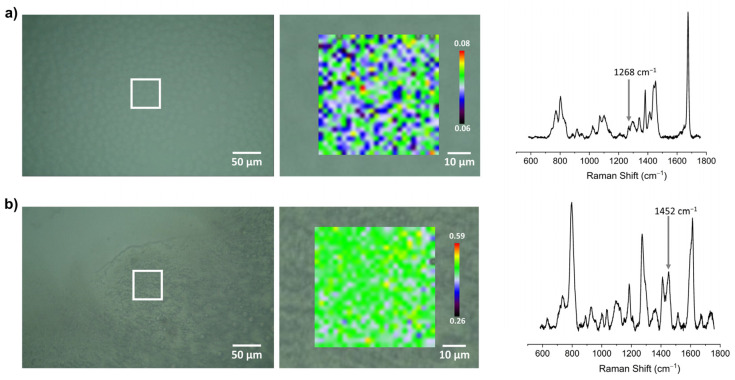
Raman maps of Z-E-50 (**a**) and Z-L-50 (**b**) illustrating distribution of the characteristic functional groups (with corresponding optical micrographs in the background) and spectra of selected regions.

The distribution of functional groups (eugenol or linalool derivatives) in Z-E-50 and Z-L-50 was studied with Raman spectroscopy mapping (Figure 6). Hybrid samples Z-E-50 and Z-L-50 were coated on glass plates by a drop casting technique (the layer prepared with dip coating was too thin to collect spectra of sufficient quality). The position of characteristic Raman shifts was correlated with the literature data [50,66,67,68]. Comparative analysis of Raman spectra of the components (Z-Me-100, Z-E-100 and Z-L-100) and hybrid blends (Z-E-50 and Z-L-50) showed that many characteristic peaks partially overlap. Based on the literature data, we selected characteristic peaks at 1268 cm^−1^ (νC_ar_-O) and 1452 cm^−1^ (δCH_2_) to map eugenol and linalool residues, respectively. In both cases, a rather uniform distribution of E and L residues was found, despite the fact that the hybrid sol was prepared not by mixing trialkoxysilanes MeSi(OEt)_3_ and E-Si(OEt)_3_ or L-Si(OEt)_3_ before their hydrolysis but by combining synthesized hydrolyzate components separately.

#### 3.2.2. Contact-Angle Measurements

**Figure 7:**
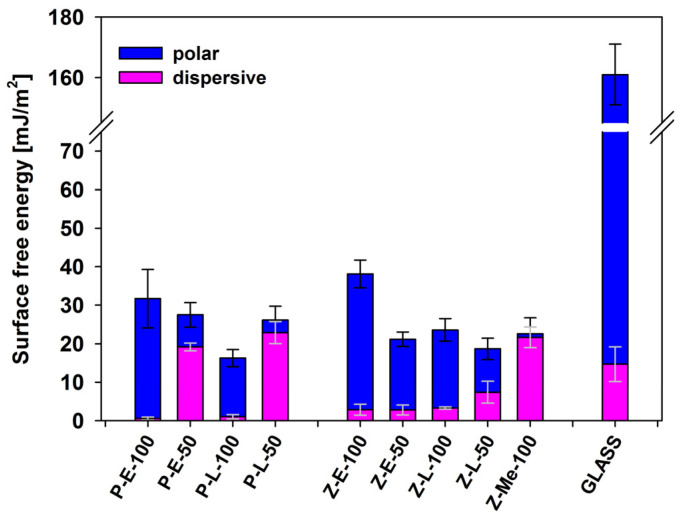
Surface free energy of functionalized polysiloxanes and sol-derived surfaces in comparison to the bare glass support (data presented as the mean ± standard deviation (SD, n = 4)).

Wettability of all tested surfaces was determined by the contact-angle measurements (sessile drop technique) using water and glycerol as reference liquids. The values of the contact angles (standard deviation estimated) are given for a short contact time, strictly observed to reduce the effect of surface accommodation. The surface free energies and their polar and dispersive components were estimated by the Owens–Wendt method, using the values of advancing angles (Figure 7). It was found that the wettability of polymer-coated glass plates decreased significantly (regardless of the coating method) when compared to that of a pure glass substrate. In general, coating glass with both linear polysiloxanes and oligosilsesquioxane sols reduced the surface free energy about five times. The overall surface free energy of the coatings is similar to that of hydrophobic Z-Me-100.

The coating method was also found to have a negligible effect on overall surface wettability. The surface energy of P-type materials applied with a slit applicator is similar to that of Z-type surfaces coated by immersion in sol solutions. However, significant differences in the ratio between polar and dispersive components between P-E-50/Z-E-50 and P-L-50/Z-L-50 were noted. As expected, the dispersive component in the surface energy of copolymer coatings (P-E-50 and P-L-50) is much larger than the polar constituent. The latter predominates for P-E-100 and P-L-100 that have a relatively larger number of hydroxyl groups. In contrast, a significant content of the polar component is characteristic for all Z-type coatings, irrespective of the number of active groups. Since the spectroscopic studies we conducted for Z-E-50 and Z-L-50 did not show heterogeneous distribution of the functional groups in the case of Z-E-50 and Z-L-50, then this observation can be explained by a different arrangement of molecules in the coating layer. Therefore, the differences between the surface characteristics of P- and Z-type samples should rather be attributed to the fact that the sol coatings are covalently bonded to the glass surface. Therefore, bioactive molecules grafted to Si atoms are forced to head towards the interface with air, while the linear polysiloxanes of a significant degree of chain mobility can adopt the most energetically favorable chain conformation to minimize surface energy.

### 3.3. Antibiofilm Properties of the Functionalized Silicones

#### 3.3.1. Antimicrobial Activity of Compounds Used in the Synthesis of Functional Polymers

**Figure 8:**
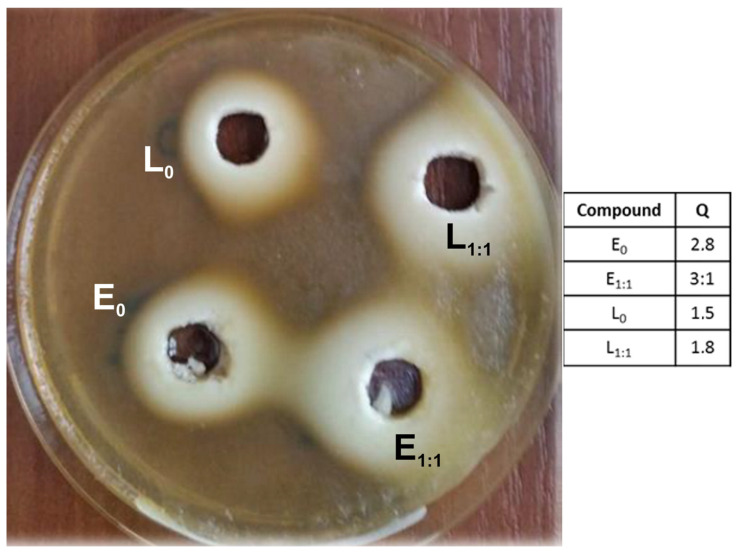
*Aeromonas hydrophila* growth inhibition zones induced by linalool and eugenol: eugenol, E_0_; linalool, L_0_—not diluted; eugenol, E_1:1_; linalool, L_1:1_—diluted with water in a ratio of 1:1.

The active compounds linalool and eugenol, as well as the functionalized polysiloxane materials, were tested against water-born bacterial strain *Aeromonas hydrophila* a KC756842 with strong adhesive properties [56]. The tested strain showed a high sensitivity to both compounds used (Figure 8).

The obtained results indicate that both tested alcohols showed antibacterial properties, but slightly higher activity expressed by the Q coefficient equal 2.8 (undiluted) and 3.1 (diluted 1:1) were obtained for eugenol. The higher Q values for aqueous solutions of tested compounds are not surprising. The addition of water improves the antimicrobial action of many sanitizers, e.g., alcohols or chitosan. The potent antibacterial activity is due to structural organization of antimicrobials, allowing an increase in the contacting regions with the bacterial surfaces and better penetration in aqueous solutions [69].

The antibacterial effect of linalool and eugenol was presented in numerous studies. Lahiri and co-workers documented that these compounds were found to be active against *Pseudomonas aeruginosa* cells [70]. A similar study was conducted against bacteria from the genus *Cronobacter*, but a slightly better effect was obtained for eugenol [71].

Recent updates on bioactive properties of linalool suggest that linalool—aromatic monoterpene alcohol—exerts antimicrobial effects through disruption of cell membranes, and its antibiofilm action is a result of blocking the process of bacterial communication or the synthesis of QS signaling molecules—N-acylhomoserine lactones [72]. Eugenol also has a similar effect and can damage the cell membranes, leading to leakage of cytoplasmic contents [73]. In addition, this compound shows wide range activity against fungi and bacteria. The study also reports the effects of eugenol on multi-drug resistant microorganisms [70]. Therefore, taking into account our own research results and the literature data, it could be assumed that linalool and eugenol would show the anti-adhesive effect of the created functional polymers against adhesive Gram-negative bacteria *A. hydrophila*.

#### 3.3.2. Anti-adhesive Properties of the Functionalized Silicones

Our experience in testing the adhesion of bacteria to various abiotic carriers prompted us to conduct long, 6-day experiments with the tested polymers and bacterial strain. This strategy is suitable for testing the anti-adhesive activity of a polymer surface, and it allows for evaluation of the reaction of microbial cells to functional groups grafted on the polymer materials. The rapid and simple luminometric method allows the estimation of biological material on the test surfaces. This method is based on bacterial ATP quantification and can be used not only to indirectly evaluate the number of adhered cells, but also their vitality, including cells that are unable to grow. A similar methodology was used in our previous studies on bioactive polymers [71]. Based on our previous experience, it can be concluded that all the functionalized silicones may show strong antimicrobial activities; however, their action depends on both the type of material and content of active groups. This may explain the direction of our current research, employing the methodology previously used and proven in this type of research. The results of luminometric and microscopic tests should be reported together.

**Figure 9:**
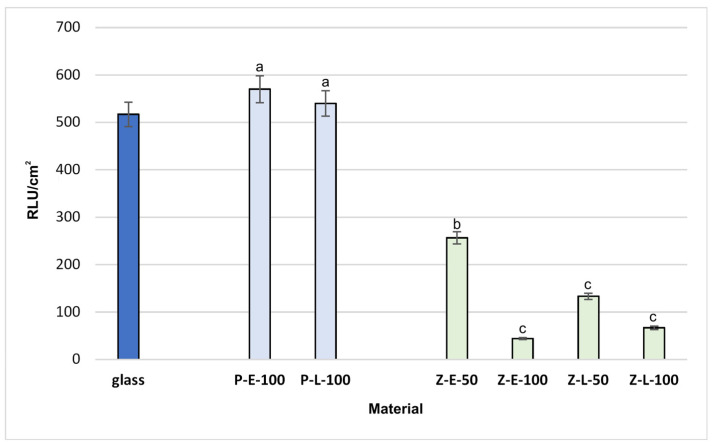
Adhesion of *Aeromonas hydrophila* to glass (control) and the functionalized silicones. The results were compared to those for the control glass carrier. Values with letters show statistically significant differences: a, *p* ≥ 0.05; b, 0.005 < *p* < 0.05; c, *p* < 0.005.

Figure 9 presents the cell adhesion on the selected modified materials after 6 days of incubation, expressed as the RLU/cm^2^. There is a striking difference in the adhesion of bacterial cells on the surfaces of Z-type in comparison to the P-type model. For the tested bacterial strain, the adhesion in the presence of functionalized silicones Z-E-100 and Z-L-100 was even 10 times lower in comparison to the control glass sample and silicones P-E-100 and P-L-100. It can be assumed that the higher RLU results for P-E-100 or P-L-100 surfaces may be due to the higher stickiness of these materials than the sol coatings, which may have stimulated the attachment of not only bacterial cells, but also organic matter from the culture medium. Contrary to the trend observed for polymer coatings, the higher number of active groups on functionalized polysilsesquioxane coatings resulted in an increase in the anti-adhesive activity of Z-E-100 and Z-L-100 compared to Z-E-50 and Z-L-50, respectively.

**Figure 10:**
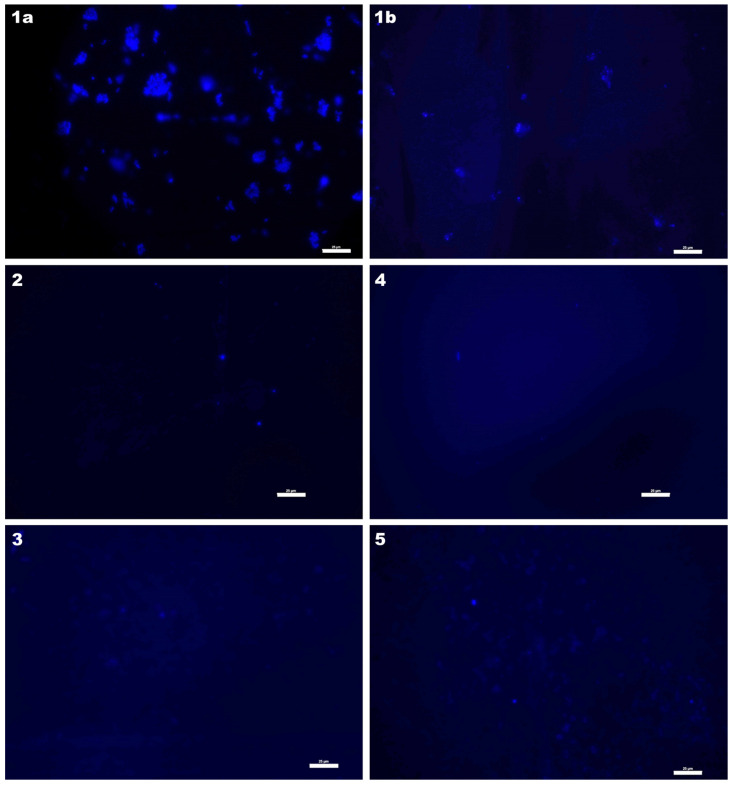
Cell attachment observed by fluorescence microscopy: (**1a,1b**) control; (**2**) P-E-100; (**3**) P-L-100; (**4**) Z-E-100; (**5**) Z-L-100 (scaling bar: 25 µm).

Bacterial adhesion was also studied by microscopic observations which were not fully confirmed by the results of the luminometric tests (Figure 10). The results of bioluminescence indicated that P-type surfaces do not exhibit anti-adhesive or antimicrobial activity towards tested bacteria. However, the microscopic image of these carriers proves something else. We suppose that the higher results for the RLU surface for P-type carriers resulted from the possible adhesion of organic compounds from the culture medium so-called “surface conditioning”, because this type of carrier (P) was characterized by much higher viscosity than Z-types. In the case of Z-type carriers, there is no such contradiction between the results. However, it needs to be emphasized that carriers of this type were characterized by definitely different surface properties.

Bacterial cells on the glass surface (control) were clustered, possibly due to uninterrupted formation of extracellular polymeric substances (EPS). On the other hand, on the surfaces modified with linalool or eugenol, cells appeared sparse and scattered. The obtained results are very interesting as they suggest that not only the type of bioactive groups but also the type of matrix and the method of biocide application may significantly affect the antimicrobial and anti-adhesive nature of the surface. Similar results were obtained for other materials, especially useful in medical applications [72,73].

RLU scores for P-type surfaces after a 6-day incubation were high, indicating adherent organic matter rather than the presence of active bacterial cells. This can be assumed because microscopic images do not show numerous bacterial cells on these materials. On the other hand, photos of the control glass sample show that a high RLU value is obtained by bacterial cells arranged in clusters, the number of which is much greater than on P-type carriers. Different conclusions can be drawn by analyzing the results of two tests for Z-type surfaces. For these materials, both RLU values and microscopic images prove anti-adhesive and antimicrobial properties. The 6-day incubation time of the carriers was sufficient for the formation of biofilm in any possible form—clusters or continuous layers. Thus, the absence of such cellular structures after incubation period certainly proves the antibacterial activity of both types of materials (P-type and Z-type), and in the case of Z-type carriers, anti-adhesive properties as well.

## 4. Conclusions

Hybrid polymeric organosilicon materials functionalized by grafting eugenol or linalool were obtained via Pt_2_(dvs)_3_ catalyzed hydrosilylation of the bioactive phytochemicals with linear polysiloxanes and triethoxysilane. The triethoxysilane derivatives were then used in the sol–gel process as precursors of oligomeric silsesquioxanes. Antibiofilm activities of the coatings were tested against bacteria *Aeromonas hydrophila*. Despite similarities in the chemical structure and surface free energy, the type of the organosilicon material proved to be a significant factor affecting the coating properties as well as the overall antibiofilm activity score.

Non-tacky coatings prepared from functionalized oligosilsesquioxanes proved to be highly anti-adhesive. A clear relationship was observed between the type and the amount of active groups as well as the antibiofilm properties of silsesquioxane materials (up to about 4x reduction of bacterial cell adhesion for hybrid coating Z-L-50 and 8× for Z-L-100 vs., respectively, 2× and 12× for the eugenol analogs). The obtained polysilsesquioxane coatings based on natural active compounds of plant origin are thus both an effective and safe alternative to conventional materials with antibiotics. The encouraging results achieved with silsesquioxane coatings are noteworthy, as this is the first example of polysilsesquioxanes grafted with linalool.

Regardless of the good film-forming properties of functionalized linear polysiloxanes, their surface was much tackier, which could stimulate the attachment of some organic matter from culture media and consequently significantly reduce the anti-adhesion properties. It corroborates previous reports on the influence of surface mechanical properties on bacterial colonization. Although the functionalized polysiloxanes did not show the expected antibiofilm properties, they can be used as models for studies on differences in chemical and thermal properties of silicone materials grafted with eugenol and linalool.

## Figures and Tables

**Table 1:** Thermal degradation characteristics of the functionalized polysiloxanes and their precursors.

Sample	Residue [%]	Residue Calculated for SiO_2_ [%]	T_d5%_ [^o^C]	T_d1_ [^o^C]
P-H-100	11.1	0.0	159.3	276.3
P-H-50	1.1	44.8	135.7	313.5
Eugenol	0.4	-	72.9	122.2
P-E-100	35.9	26.7	336.0	433.8
P-E-50	23.9	20.10	308.8	449.0
Linalool	0.0	-	45.1	87.7
P-L-100	0.0	28.0	244.1	276.7
P-L-50	0.6	20.8	241.9	290.6

## Data Availability

Data presented in this study are available on request.

## Supplementary Materials

The following supporting information can be downloaded at: https://www.mdpi.com/article/10.3390/ma16041381/s1, Scheme S1: Chemical structure assignment of the synthesized organosilicon derivatives of eugenol and linalool; Figure S1a: ^1^H NMR spectra of linear polysiloxanes grafted with eugenol (compared to their precursors P-H-100 and P-H-50); Figure S1b: ^1^H NMR spectra of linear polysiloxanes grafted with linalool (compared to their precursors P-H-100 and P-H-50); Figure S2a: ^13^C NMR spectra of linear polysiloxanes grafted with eugenol (compared to their precursors P-H-100 and P-H-50); Figure S2b: ^13^C NMR spectra of linear polysiloxanes grafted with linalool (compared to their precursors P-H-100 and P-H-50); Figure S3: ^1^H NMR spectra of the products obtained by hydrosilylation of linalool (a) and eugenol (b) with HSi(OEt)_3_; Figure S4: ^13^C NMR spectra of the products obtained by hydrosilylation of linalool (a) and eugenol (b) with HSi(OEt)_3_; Figure S5: FT-IR absorbance spectra of linear polysiloxanes grafted with eugenol and linalool (compared to their precursors P-H-100 and P-H-50); Figure S6: FT-IR absorbance spectra of spectra of the products obtained by hydrosilylation of linalool and eugenol with HSi(OEt)_3_; Table S1: Assignment of peaks in IR spectra of functionalized organosilicon polymers and monomers.

## Sample Availability

Samples of the compounds are available from the authors only for scientific collaboration.
