# Supplementary material for: Hybrid Bio-Based Silicone Coatings with Anti-adhesive Properties
Source: Materials (Basel). 2023 Feb 7;16(4):1381. doi: 10.3390/ma16041381 (PMC9961570; doi:10.3390/ma16041381)
Supplement: Supplementary file 1 [file materials-16-01381-s001.zip › materials-2111242-supplementary.pdf]

Hybrid bio-based silicone coatings with antiadhesive properties

Supporting Information

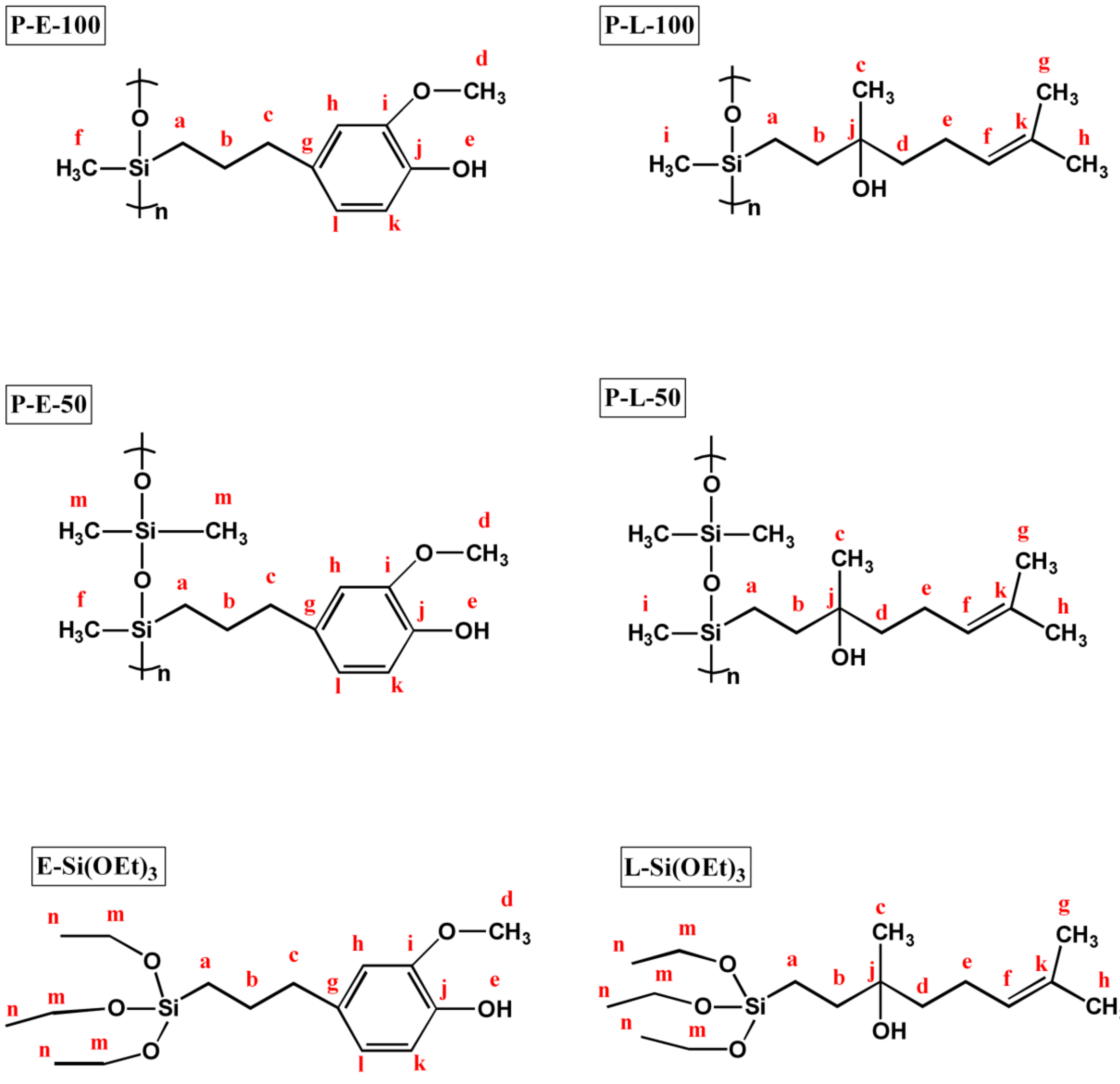

Scheme S1. Chemical structure assignment of the synthesized organosilicon derivatives of eugenol and linalool.

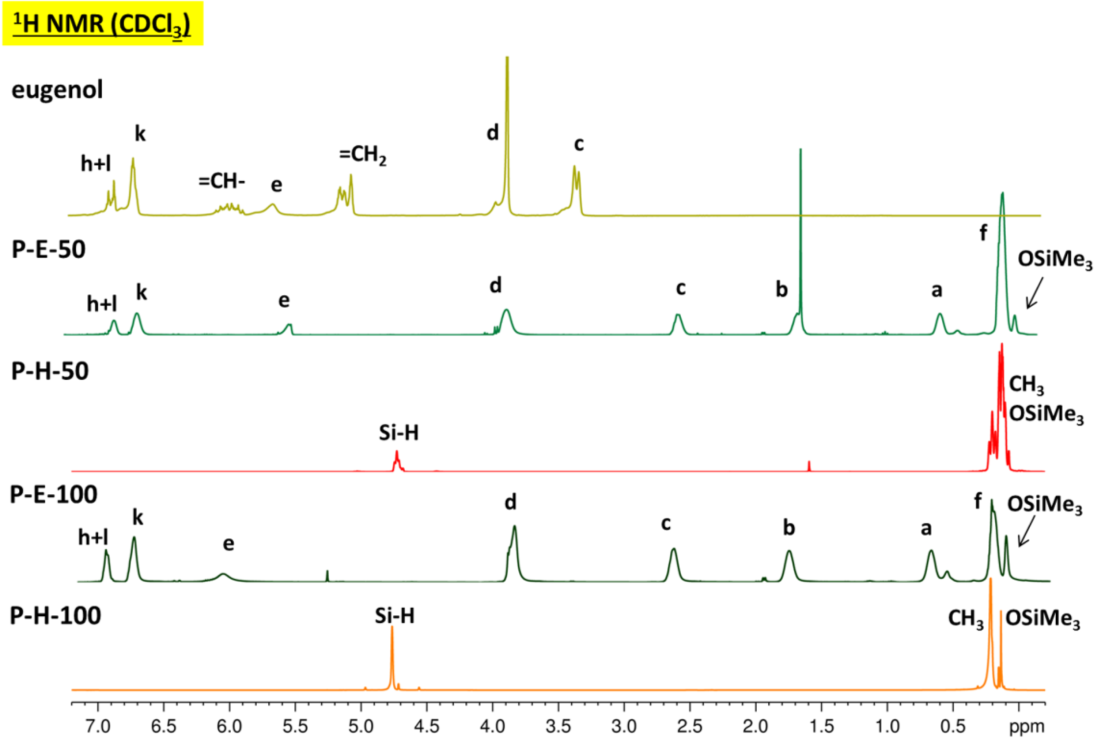

(a)

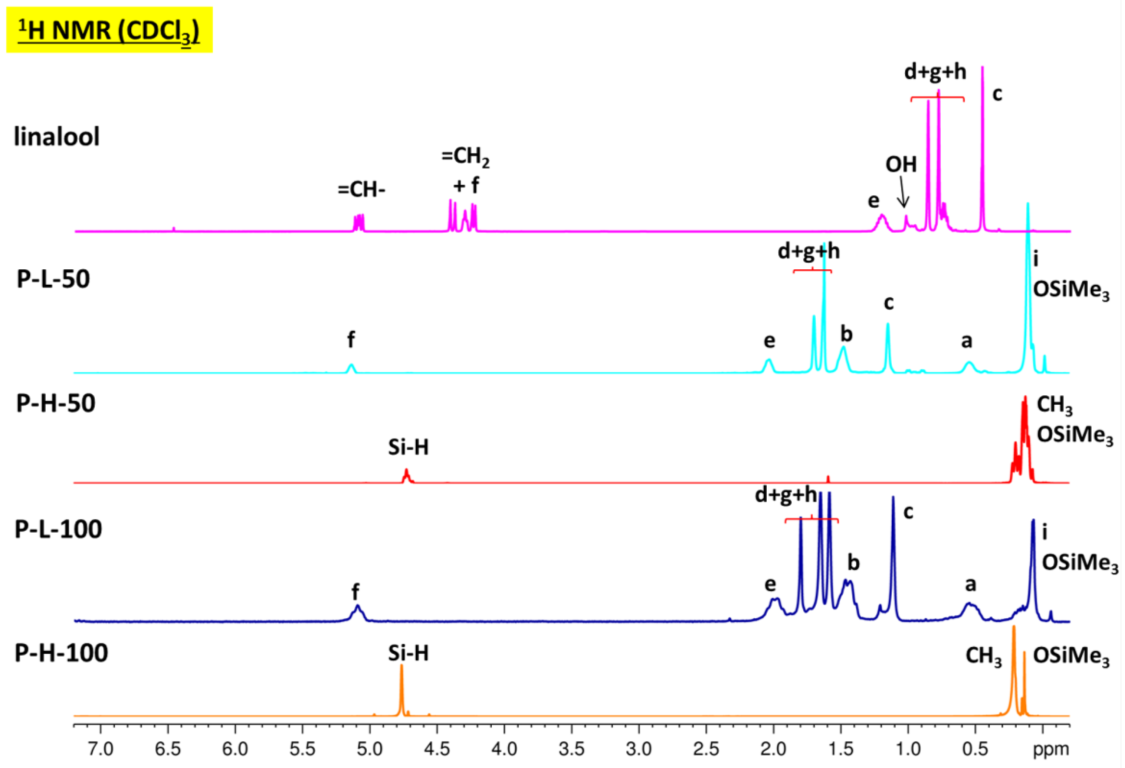

(b)

Figure S1. (a)^{1}H NMR spectra of linear polysiloxanes grafted with eugenol (compared to their precursors P-H-100 and P-H-50). **(b).** ^{1}H NMR spectra of linear polysiloxanes grafted with linalool (compared to their precursors

P-H-100 and P-H-50).

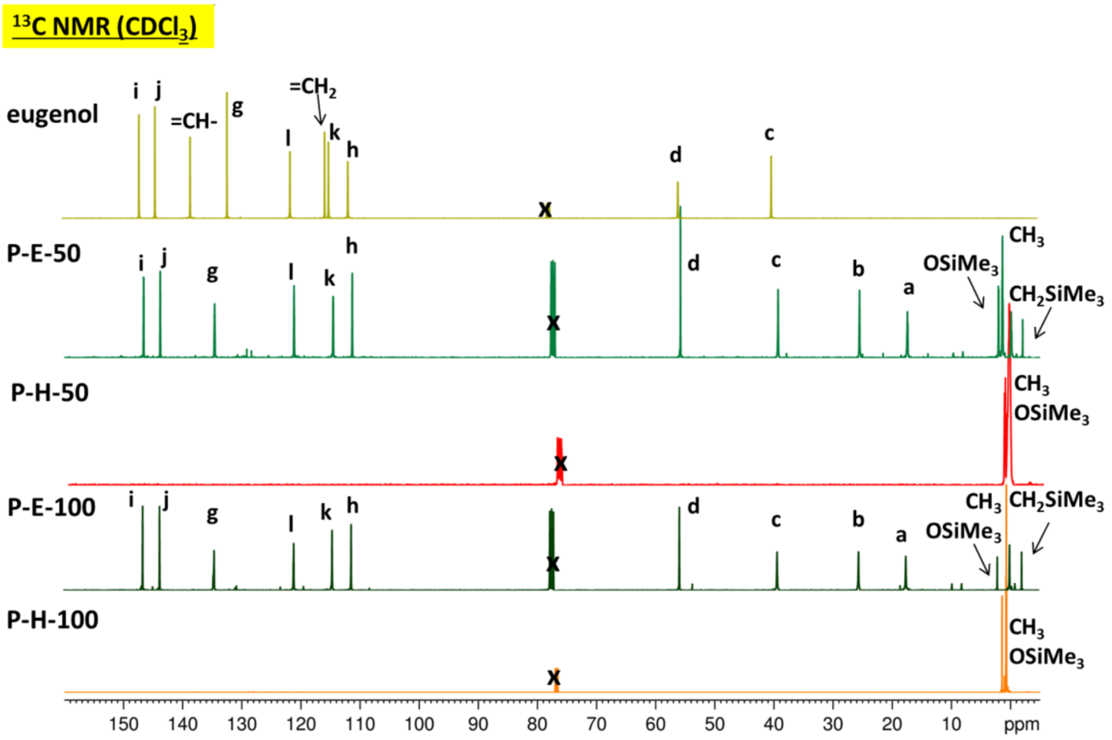

(a)

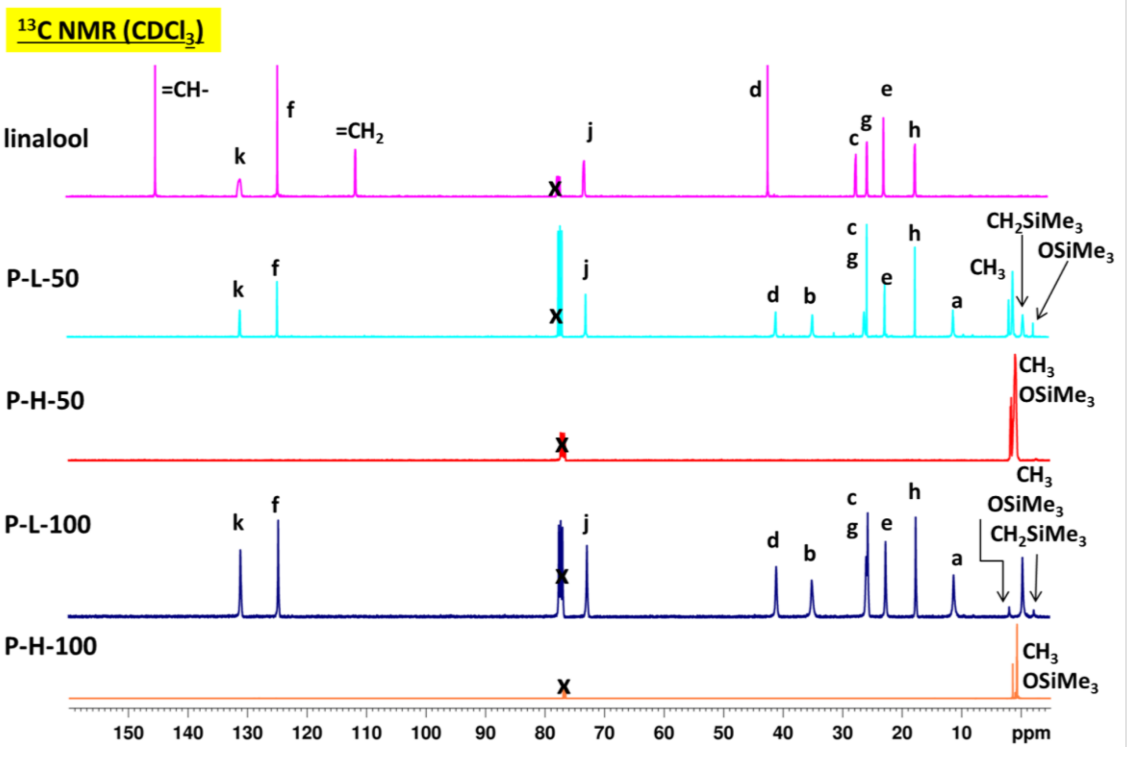

(b)

Figure S2. (a). ^{13}C NMR spectra of linear polysiloxanes grafted with eugenol (compared to their precursors P-H-100 and P-H-50). **(b).** ^{13}C NMR spectra of linear polysiloxanes grafted with linalool (compared to their precursors P-H-100 and P-H-50).

^1H NMR ($CDCl_3$)

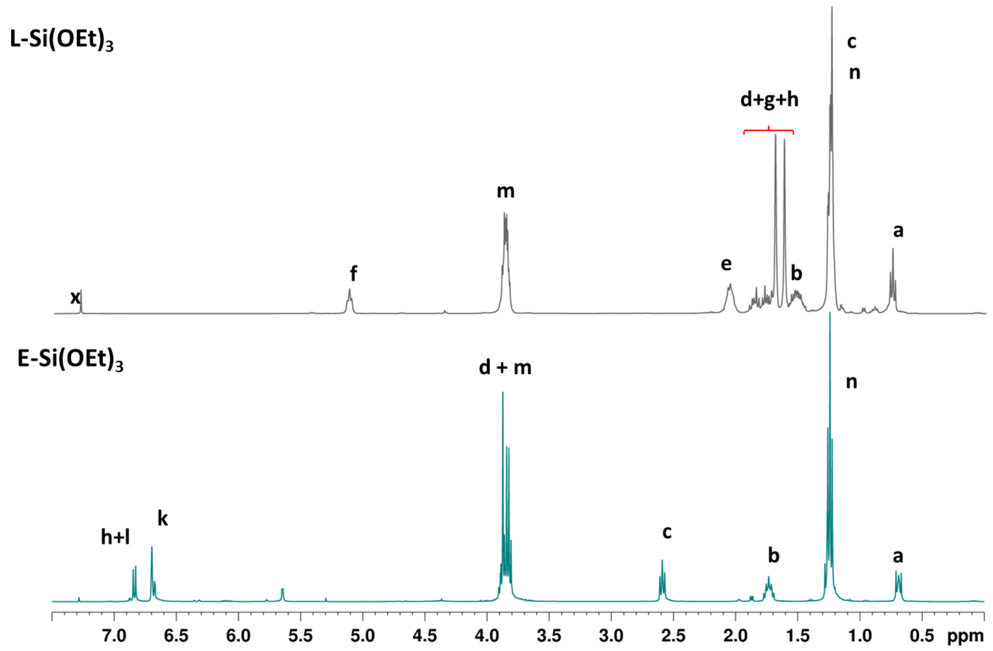

Figure S3. ^1H NMR spectra of the products obtained by hydrosilylation of linalool (a) and eugenol (b) with $HSi(OEt)_3$.

^{13}C NMR ($CDCl_3$)

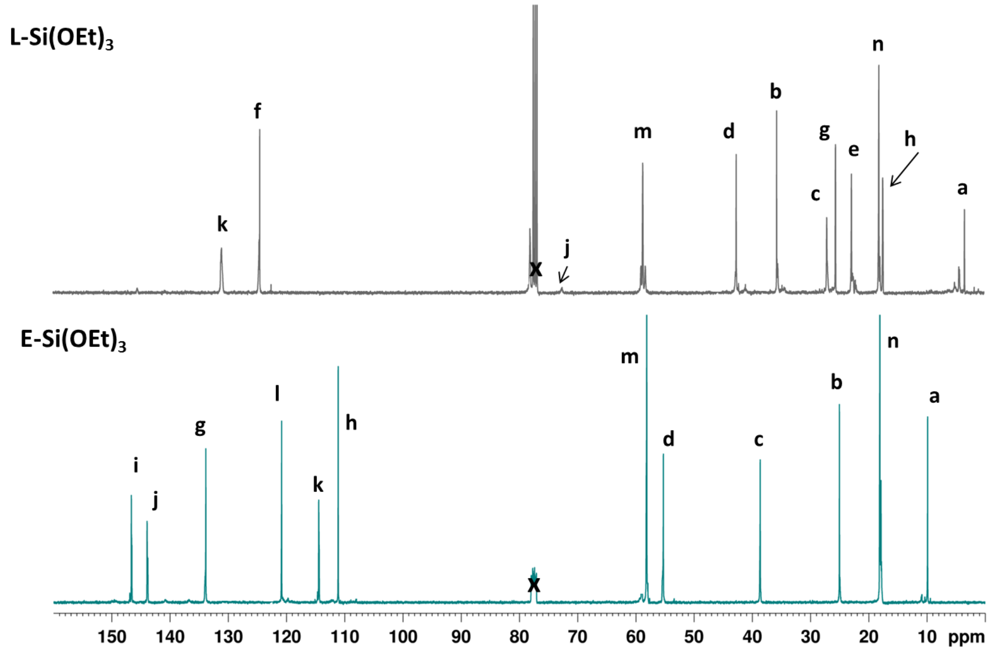

Figure S4. ^{13}C NMR spectra of the products obtained by hydrosilylation of linalool (a) and eugenol (b) with $HSi(OEt)_3$.

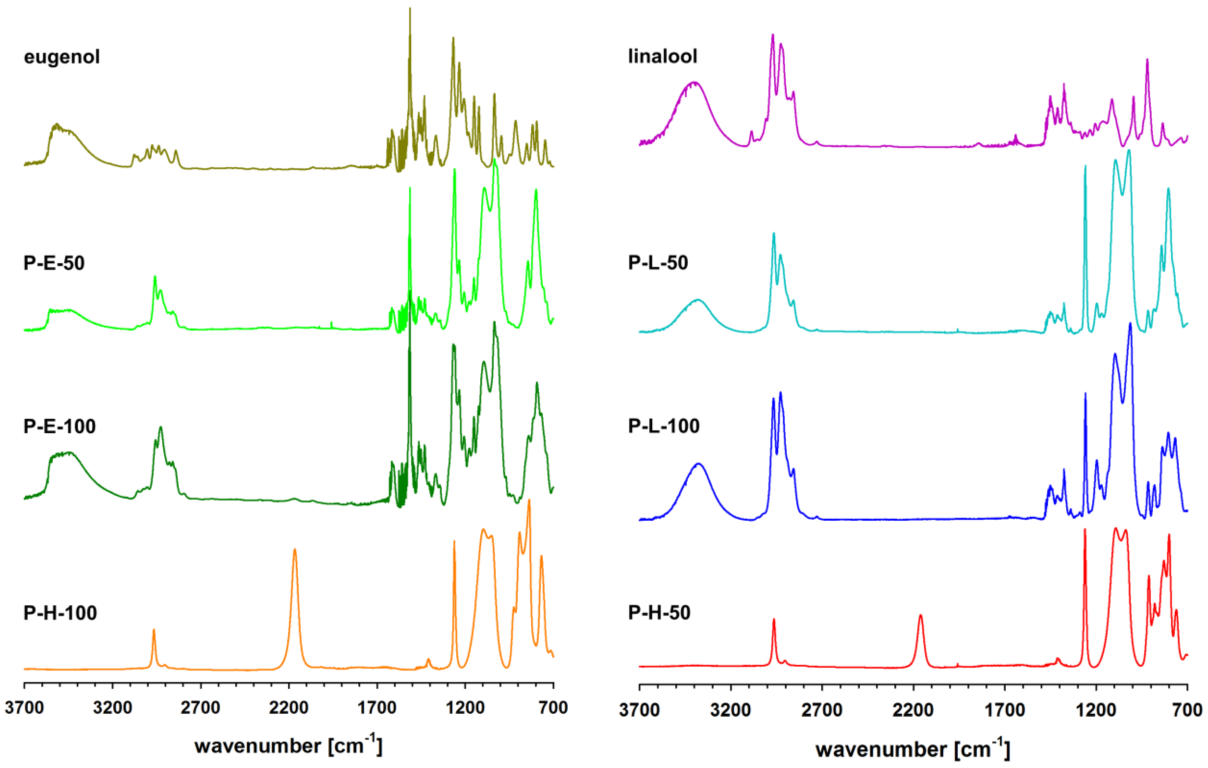

Figure S5. FT-IR absorbance spectra of linear polysiloxanes grafted with eugenol and linalool (compared to their precursors P-H-100 and P-H-50).

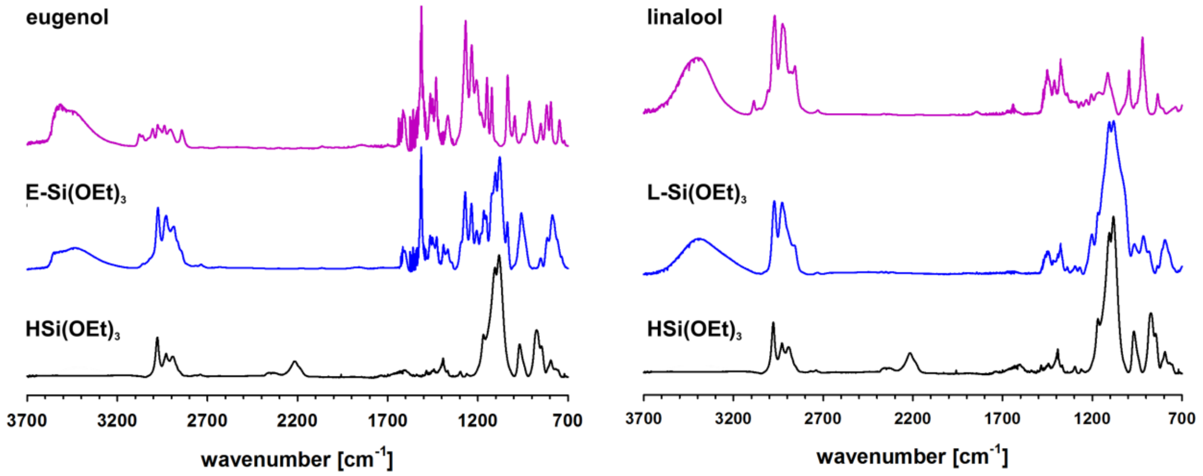

Figure S6. FT-IR absorbance spectra of spectra of the products obtained by hydrosilylation of linalool and eugenol with $HSi(OEt)_3$.

Table S1. Assignment of peaks in IR spectra of functionalized organosilicon polymers and monomers.

	wavenumber [cm^{-1}]					wavenumber [cm^{-1}]			
**vibration**	**Eugenol**	**P-E-100**	**P-E-50**	**E-Si(OEt)$_3$**	**vibration**	**Linalool**	**P-L-100**	**P-L-50**	**L-Si(OEt)$_3$**
ν(OH)	3580-3260	3600-3260	3580-3270	3580-3240	ν(OH)	3640-3180	3580-3180	3600-3100	3600-3120
ν(C-H)	3100-2810	3040-2810	3050-2830	3050-2790	ν(C-H)	3040-2780	2990-2820	2962-2860	3000-2840
ν(C=C)$_{arom}$	1609	1609	1613	1613	ν(CH=CH$_2$)	1642	-	-	-
ν(C=C)$_{ring}$	1511	1507	1511	1511					
ω(CH$_3$), δ$_s$(CH$_2$)	1434	1434	1421	1421	ω(CH$_3$), δ$_s$(CH$_2$)	1446	1458	1471	1446
					ω(C-H)$_{Vi}$	1413	1413	1422	1417
δ(COH)	1372	1368	1364	1364	δ(COH)	1381	1372	1373	1377
δ Si-CH$_3$	-	1266	1262	-	δ Si-CH$_3$	-	1258	1258	-
ν(C$_{arom}$-O-CH$_3$)	1237	1229	1238	1238					
δ Si-CH$_2$	-	1151	1151	1152	δ Si-CH$_2$	-	1196	1197	1201
ν(Si-O-Si)	-	1095<br>1035	1094<br>1033	-	ν(Si-O-Si)	-	1098<br>1017	1107<br>1022	-
ν(Si-OEt)	-	-	-	1102<br>1082	ν(Si-OEt)	-	-	-	1107<br>1078
τ(C=C)	996	-	-	-	τ(C=C)	1000	-	-	-
ω(=CH$_2$), ρ(=CH$_2$)	911	-	-	-	ω(=CH$_2$), ρ(=CH$_2$)	919	919	914	915
ν(C-C) in Si-OEt	-			960	ν(C-C) in Si-OEt	-	-	-	964
ρ Si-CH$_3$	-	792	794	790	ρ Si-CH$_3$	-	804	804	796
